# Do Preschool Children Learn to Read Words from Environmental Prints?

**DOI:** 10.1371/journal.pone.0085745

**Published:** 2014-01-22

**Authors:** Jing Zhao, Pei Zhao, Xuchu Weng, Su Li

**Affiliations:** 1 Key Laboratory of Behavioral Science, Institute of Psychology, Chinese Academy of Sciences, Beijing, China; 2 Center for Cognition and Brain Disorders, Hangzhou Normal University, Hangzhou, China; 3 Zhejiang Key Laboratory for Research in Assessment of Cognitive Impairments, Hangzhou, China; Hamamatsu University School of Medicine, Japan

## Abstract

Parents and teachers worldwide believe that a visual environment rich with print can contribute to young children's literacy. Children seem to recognize words in familiar logos at an early age. However, most of previous studies were carried out with alphabetic scripts. Alphabetic letters regularly correspond to phonological segments in a word and provide strong cues about the identity of the whole word. Thus it was not clear whether children can learn to read words by extracting visual word form information from environmental prints. To exclude the phonological-cue confound, this study tested children's knowledge of Chinese words embedded in familiar logos. The four environmental logos were employed and transformed into four versions with the contextual cues (i.e., something apart from the presentation of the words themselves in logo format like the color, logo and font type cues) gradually minimized. Children aged from 3 to 5 were tested. We observed that children of different ages all performed better when words were presented in highly familiar logos compared to when they were presented in a plain fashion, devoid of context. This advantage for familiar logos was also present when the contextual information was only partial. However, the role of various cues in learning words changed with age. The color and logo cues had a larger effect in 3- and 4- year-olds than in 5-year-olds, while the font type cue played a greater role in 5-year-olds than in the other two groups. Our findings demonstrated that young children did not easily learn words by extracting their visual form information even from familiar environmental prints. However, children aged 5 begin to pay more attention to the visual form information of words in highly familiar logos than those aged 3 and 4.

## Introduction

A vast amount of research in the past decades has shown that early literacy experience and preliteracy knowledge can predict later literacy outcomes [1], [2], [3], [4]. Based on the perspective of “emergent literacy”, the early phases before children read and write conventionally have been considered as a crucial period for school reading readiness [5], [6]. Consequently, early childhood educators, teachers and parents have given increasing attention on how to foster children's preliterate knowledge and skills so that they can provide a foundation for later learning to read.

Reading to children each day is one of the most beneficial ways in which a parent can promote literacy. Picture book reading, especially shared book reading has been advocated as an important activity to promote children's language and literacy skills during the preschool years [7], [8]. Also, educators and teachers employ narrative conversations to prompt preschool children's vocabulary [9], [10]. Indeed these activities have been shown to have positive effects on children's development of language and literacy [4], [11], [12], [13], [14].

Parents and teachers regard environmental experiences as important influence on early literacy development as well. Therefore, they create a rich print environment in children's daily life, such as providing child with alphabet fridge magnets, alphabet blocks, and word cards with corresponding pictures on it. They expect that the vast exposure to prints may facilitate children's word reading before they enter into primary school. Kindergarten teachers often put some product labels, such as Sesame Street, and McDonalds, which children known, onto the walls in classroom to help them learn words in logos. However, it is unclear as to whether these environmental prints are indeed helpful for young children to learn the words contained in the logos. Limited evidence exists regarding whether children treat the environmental prints simply as pictures or pictorial symbols or they are able to extract the visual word form information from the logos.

Some research investigated the aforementioned issue by measuring children's ability to read words contained in various versions of environmental prints [15], [16], [17]. In these studies, preschool children were presented with either familiar logos (e.g., McDonald's), or the same logos with their color, image or font type cues removed, and asked to name words contained in the various versions. Results showed that although children's naming accuracy became poorer as these contextual cues were removed, they still showed some recognition of the words [15], [17]. The finding suggested that preschool children seemed to be able to extract visual word information from familiar logos. Consistent with this idea, a training study found that preschool children more quickly learned to read words from environmental prints (e.g., McDonald's) than control words (e.g., Monster); despite all these words were transformed into printed versions [18]. This finding again suggested that young children might have some implicit knowledge about the visual word form information of environmental prints.

It is important to note that the previous findings that children appeared to learn visual word form information from familiar logos were mostly from the studies conducted in children who learn to read alphabetic scripts. However, in alphabetic scripts, letters regularly correspond to phonological segments in a word and provide strong cues about the identity of this word [19]. Thus, it is possible that children have learned the letters elsewhere and applied them to guess correctly the identity of some of words in familiar logos. If this was the case, it would be questionable as to whether previous findings indeed suggested that young children have extracted visual word form information directly from environmental prints.

To control for this major confound, one needs to use word scripts without direct grapheme to phoneme mapping such that children cannot use the sound gleaned from part of the word to guess its identity. This issue is typically difficult to resolve in most of the alphabetic written languages. In this regard, the Chinese written system has a unique advantage. Linguistically, the visual form of a Chinese character provides limited information about the sound form of this character, and there is essentially no grapheme to phoneme conversion [20], [21]. A Chinese character maps onto phonology at the syllable level, without any parts in a character corresponding to phonemes. Moreover, the mapping between the visual form and its phonology is relatively arbitrary in some characters, especially characters young children encounter often. The characteristics of Chinese written system thus allow us to test whether young children are able to learn words by extracting their visual form information from environmental prints.

The present study tested Chinese preschool children's knowledge of Chinese words embedded in highly familiar environmental logos. With the removal of the phonological-cue confound, we aimed at examining specifically whether preschool children could learn words by extracting their visual form information from highly familiar environmental logos. We used four logos with which most preschool children are highly familiar (e. g., Ken3 De2 Ji1, the Chinese logo of KFC). To examine the extent to which preschoolers could recognize the words from exposure to the environmental prints, in addition to the logos in their original format, each logo was transformed into three versions with familiar contextual cues gradually minimized. In specific, there were four versions of these logos: (a) a logo in its actual color (i.e., *a colorized logo*); (b) a black-and-white photocopy of the actual logo (i.e., *a black-and-white logo*, the *color* cue was removed); (c) a black-and-white photocopy of the words with its original font but without an accompanying picture (i.e., *words with the original font*, both *color* and *logo* cues were removed) and (d) words in a printed font without any cues (i.e., *words in printed font*, the *color*, *logo* and *font* type cues were all removed). In addition, most of the previous studies only tested children in one age group [18] or in a mixed-age group [15], [16]. However, the role of various cues of environmental prints in children learning words may change with age since learning to read is a developmental process. We thus systematically tested children at different ages (3, 4 and 5 years). They were asked to name the words contained in the stimuli that were either contextualized (a, b, c) or de-contextualized (d). If children are able to learn words by extracting the visual word form information from environmental prints like conventional word reading, they would perform well even in the de-contextualized version. Alternatively, if children read words depending on contextual cues, they would perform better when the words with contextual cues than de-contextualized. And further, if the role of various cues changes with age, different aged children would perform differently when these cues are removed.

## Methods

### Ethics Statement

All of the parents or guardians of the children gave written, informed consent in accordance with procedures and protocols approved by the human subjects review committee of the Institute of Psychology, Chinese Academy of Sciences.

### Participants

Ninety-two kindergarten children were tested. All participants were native Chinese speakers with normal or corrected-to-normal vision. Thirty were 3-year-olds (M_age_ = 3.61 years, SD = 0.28, 16 males), thirty-two were 4-year-olds (M_age_ = 4.58 years, SD = 0.21, 14 males), and thirty were 5-year-olds (M_age_ = 5.69 years, SD = 0.29, 18 males). They were not taught formally to learn to read words in the kindergarten. Most of children's parents earned a bachelor's degree.

### Materials

Four logos that were most familiar to preschool children were selected, including the logos of KFC, McDonald's, Hao3 Duo1 Yu2 (a brand of biscuit), and Bei3 Jing1 Huan1 Ying2 Ni3 (a sign of the Beijing Olympics). These logos were selected from 30 logos according to familiarity evaluation by 28 parents in a five-point scale (1 = neverto 5 = frequent) (see Table 1). The score of logo familiarity evaluation was analyzed by a mixed 3×4 two-way ANOVA with Age as a between-subject factor and Logo as a within-subject factor. Results with Greenhouse-Geisser correction revealed no significant main effects: Age, *F*(2, 25) = 2.07, *p*>.1 and Logo, *F*(1.56, 39)<1, *p*>.1. The interaction between Age and Logo was not significant, *F*(3.12, 39)<1, *p*>.1. These results showed that the familiarity of the four logos was the same for children of different ages. As noted before, we created four versions of each environmental print by gradually removing the contextual cues including color, logo, and font: (a) a colorized logo; (b) a black-and-white logo; (c) words with the original font; (d) words in printed font (see Introduction for the detailed manipulation of the four versions).

**Table 1:** Mean scores of familiarity evaluation.

	好多鱼	肯德基	麦当劳	北京欢迎你
3-year-old	5.00 (0.00)	4.56 (1.33)	4.67 (0.71)	5.00 (0.00)
4-year-old	4.33 (1.12)	4.56 (1.01)	4.56 (0.73)	4.56 (1.33)
5-year-old	5.00 (0.00)	5.00 (0.00)	5.00 (0.00)	5.00 (0.00)

*Note:* 好多鱼-Hao3 Duo1 Yu2; 肯德基-KFC; 麦当劳-McDonalds; 北京欢迎你-Bei3 Jing1 Huan1 Ying2 Ni3; Score 5 indicates the logo most frequently appearing in the children's surrounding. Standard deviations of means are given in parentheses.

### Procedure

The children were individually tested in a quiet room in the kindergarten. Children were presented with each card containing different versions of each logo. They were asked to name the words in the card (“What do the words say?”). The answers were recorded verbatim. To control the effect of the presentation order, both the order of the four versions and the four items in each version were all randomized across participants.

### Coding and reliability

The children received a score of 2 if they correctly identified the stimulus, 1 if they only provided an answer related to the logo's meaning (e.g., referring to the KFC logo as “hamburger” instead of 肯德基 (Ken3 De2 Ji1)), and 0 if they gave an incorrect answer or none at all. One primary coder recorded and coded the data from all participants and a second coder independently coded the data of 10 randomly chosen children in each group. Results showed that the kappa coefficients for each stimulus version exceeded 0.90: version a = 1.00, version b = 0.97, version c = 1.00 and version d = 1.00 (all *p*-values<.001), suggesting that the coding was reliable.

## Results

**Figure 1:**
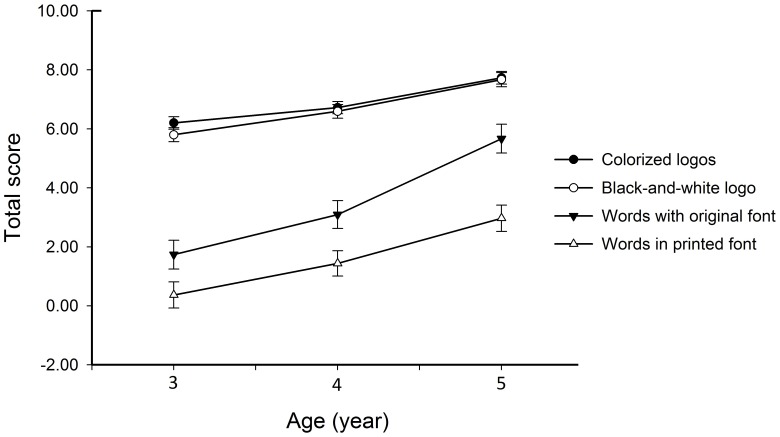
Total score for each version in children at ages of 3, 4, and 5. All three children groups performed better when words were presented with contextual cues than when they were presented in a plain fashion, devoid of context. However, the role of various cues changed with age increased as children read words contained in logos.

For each participant, the sum of scores of the four logos in each version was computed, respectively. The score ranged from 0 to 8. Figure 1 shows averaged total scores and standard errors of the four versions of environmental prints for the three children groups. Firstly, we did a 3×2×4 three-way ANOVA with Age and Gender as two between-subject factors and Version as a within-subject factor. Results with Greenhouse-Geisser correction showed that neither the main effect of Gender, *F* (1, 86) = .01, *p*>.1 nor the interaction between Gender and the other factors was significant [Gender by Age, *F* (2, 86) = 1.88, *p*>.1, Gender by Version, *F* (1.83, 157.20) = .60, *p*>.1, and Gender by Age by Version, *F* (3.66, 157.20) = 1.20, *p*>.1]. Thus, the data of boys and girls were combined in further analysis. Specifically, total score was analyzed in a mixed 3×4 two-way ANOVA with Age as a between-subject factor and Version as a within-subject factor. Results with Greenhouse-Geisser correction showed that both main effects were significant: Age, *F*(2, 89) = 24.11, *p*<.001 and Version, *F*(1.83, 162.95) = 219.22, *p*<.001. The post-hoc test further showed that children performed better when the words with full or partial contextual cues than de-contextualized (all *p*-values<.01). And the score was significantly increased with age (all *p*-values<.01). Importantly, the interaction between Version and Age was significant, *F*(3.66, 162.95) = 3.23, *p*<.05, which suggested that the role of various contextual cues changed with age as children read the words contained in the logos. In addition, the sum of scores of each item was analyzed in a 3×4 three-way ANOVA with Age and as a between-subject factors and Version as a within-subject factor. In general, the results for each logo were consistent with those for the total scores of the four logos (see also Table S1).

**Figure 2:**
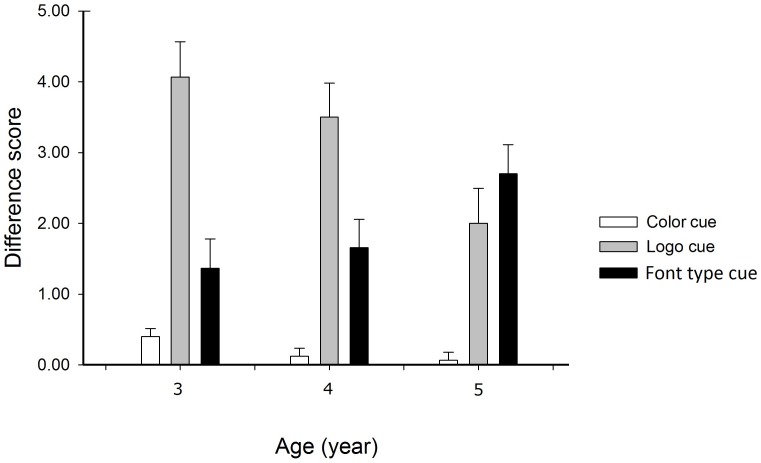
Difference scores between two versions in children at ages of 3, 4, and 5. Children at different ages showed various degrees of dependence on the cues to read words contained in environmental prints. The influence of the color cue and the logo cue was stronger in children aged 3 and 4 than those aged 5, while the role of the font type cue was stronger in the 5-year-olds than those aged 3 and 4.

As noted in the introduction, there were three types of cues: the color cue, the logo cue and the font type cue. According to the manipulation of each type of cues, we focused on the difference scores between two versions. Specifically, the role of the color cue was measured by subtracting the score of version b from that of version a. The role of the logo cue was measured by subtracting the score of version c from that of version b. And the role of the font type cue was calculated by subtracting the score of version d from score of version c. Figure 2 shows the role of different cues at different ages. We used a mixed 3×3 ANOVA with Age as a between-subject factor and Cue Type as a within-subject factor. Results with Greenhouse-Geisser correction showed that the interaction between Age and Cue Type was significant, *F*(2.60, 115.62) = 4.40, *p*<.01. To further examine the role of the cues at different ages, we broke down the two-way interaction by Cue Type. Data was analyzed in a one-way ANOVA with Age as a within-subject factor. For the color cue, results showed that the main effect of Age was marginally significant, *F*(2, 89) = 2.46, *p* = .09. The post-hoc test further showed that the role of the color cue was stronger in three-year-old children than that in four- and five-year-old children (3-year-olds vs. 4-year-olds, *p* = .09, 3-year-old vs. 5-year-old, *p*<.05), while no significant difference was observed between 4- and 5-year-old children (*p*>.1). For the logo cue, the main effect of Age was significant, *F*(2, 89) = 4.64, *p*<.05. And the post-hoc test showed that the role of the logo cue was stronger in 3- and 4- year-olds than that in 5-year-olds (3-year-olds vs. 5-year-olds, *p*<.01, 4-year-olds vs. 5-year-olds, *p*<.05), while no significant difference was found between 3- and 4- year-olds (*p*>.1). For the font type cue, the main effect of Age was marginally significant, *F*(2, 89) = 2.90, *p* = .06. The post-hoc test showed that the role of the font type cue was stronger in 5-year-olds than in the other children groups (3-year-olds vs. 5-year-olds, *p*<.05, 4-year-olds vs. 5-year-olds, *p* = .07), while no significant difference was observed between 3- and 4- year-olds (*p*>.1).

## Discussion

The present study employed the environmental logos contained Chinese characters which can better control the effect of phonological cues on visual word recognition to examine whether young children could learn words by extracting their visual form information from highly familiar environmental logos. We found that children aged 3 to 5 all performed better when words were presented in highly familiar logos compared to when they were presented in a plain fashion, devoid of context. This advantage for familiar logos was also present when the contextual information was only partial. These findings suggested that children read words in logos depending on contextual cues and verified our second hypothesis. However, the role of various cues changed with age as children learned words in the logos.

Consistent with previous studies on children who read alphabetic words [15], [16], [17], results in the present study showed that the ability of Chinese preschool children to read words in environmental prints decreased as contextual cues removed. Given that Chinese script has its special characteristics to exclude the phonological-cue confound, our findings clearly suggested that preschool children did not easily learn words by extracting their visual form information from environmental prints through passive exposure. This idea was well in accordance with findings from picture-book reading studies, which indicated that young children spent less time on words than pictures [22]. A recent study in infants also found that although some children learned from viewing an educational DVD several times a week for 4 weeks at home, this group did not learn any more words from exposure to them than did a control group [23].

Interestingly, we found an interaction between age and cue type, which indicated children at different ages, showed various degrees of dependence on different cues to read words contained in environmental prints. Younger children were affected more by the color cue and the logo cue when they read words contained in environmental prints, while the 5-year-olds were affected stronger by the font type cue. Obviously, there was a difference in the nature of the three types of cues. Specifically, the color and logo cues were not related to visual word information, while the font type cue was one aspect of the visual word form information. Our results thus suggested that children aged 5 begin to direct more attention than those aged 3 and 4 to the information of visual word form like conventional word reading. Through systematically testing the influence of various cues on children of different ages, our findings highlighted this critically transitional period in reading development. Similarly, results in a recent study on name writing in Chinese preschoolers also suggested that children began to learn about the visual properties of written Chinese from at the age of 3 to 4 and their knowledge increased fast from age 5 [24]. Thus, we provided the new evidence to determine why the 5-year-olds in the study by [18] could benefit from training in environmental print reading and why the learning effect was hardly observed in younger groups [16]. Such result also has important educational implications. Recent studies suggest the importance of providing an active and meaningful way for young children to learn words from environmental prints through positive and scaffolding interaction (see [25] for a review). Findings in the present study suggested that in order to make children get more benefits from these educational activities, teachers and parents should apply such a program into an appropriate age group consisting of children who can pay more attention to the information of visual word form.

The interaction between cue type and age was not reported in the previous studies [15], [16], [18]. One possible interpretation of this discrepancy lies in different experiment designs. The earlier studies only tested children at one age (e.g., 5 years old) [18] or in a mixed-age group [15], [16], whereas our study systematically examined children aged 3, 4 and 5. Another possibility may be differences in script features between alphabetic words and Chinese characters. As noted before, the visual word form of a Chinese character corresponds to its phonology at the syllable level without any grapheme to phoneme conversion. Visual-orthographic processing plays an especially important role for Chinese children to learn word reading [26], [27], [28]. This demand may promote them to pay more attention to visual word form with exposure to environmental prints increased. Finally, Chinese educational context such as children parents' attitude toward exposure to prints may make a contribution. Chinese parents attach great importance to children's early word reading, and some children receive extensive exposures to reading Chinese characters even before they enter primary schools. Yet the precise interpretation requires further empirical verification by testing children who read alphabetic words in a similar design used in our study. Some variables (e.g., children's literacy experience and visual word form perception competence) should be also measured. In addition, it was important to note that there were a few lines of limitation that should be considered. Firstly, to investigate the role of environmental prints in visual word reading development, logos were all highly familiar to children in the present study. It should be examined whether our findings could be generalized to less familiar logos or whether the familiarity of logos affected the findings (e.g., the effect of the font type cue in children at different ages). Secondly, the number of items in each condition was small. Our findings need to be confirmed further by using more numbers of logos in the future study. Moreover, it could be examined that whether children's performance on different items was different. Such studies may provide more evidence to understand the role of environmental prints in the development of visual word reading.

## Supporting Information

Table S1 Results of ANOVAs for total scores of each item.
(DOCX)

## References

[1] Oliver BR, Dale PS, Plomin R (2005) Predicting Literacy at Age 7 from preliteracy at Age 4: A Longitudinal Genetic Analysis. Psychological Science 16: 861–865. 16262770 10.1111/j.1467-9280.2005.01627.x

[2] Treiman R, Tincoff R, Rodriguez K, Mouzaki A, Francis DJ (1998) The foundations of literacy: Learning the sounds of letters. Child Development 69: 524–1540. 9914638

[3] Burgess SR, Hecht SA, Lonigan CJ (2002) Relations of the home literacy environment (HLE) to the development of reading related abilities: A one-year longitudinal study. Reading Research Quarterly 37: 408–426.

[4] Whitehurst GJ, Lonigan CJ (1998) Child development and emergent literacy. Child Development 69: 848–872. 9680688

[5] Sulzby E (1985) Children's emergent reading of favorite storybooks: A developmental study. Reading Research Quarterly 20: 458–479.

[6] Sulzby E, Teale W (1996) Emergent literacy. In Barr ML, Kamil PB, Mosenthal, PD Pearson, editors. Handbook of reading research. Mahway, NH: Lawrence Erlbaum. pp. 727–757.

[7] Bus AG, van IJzendoorn MH, Pellegrini AD (1995) Joint book reading makes for success in learning to read: A meta-analysis on intergenerational transmission of literacy. Review of Educational Research 65: 1–21.

[8] Dunning DB, Mason JM, Stewart JP (1994) Reading to preschoolers: A response to Scarborough and Dobrich and recommendations for future research. Developmental Review 14: 324–339.

[9] Whitehurst GJ, Falco FL, Lonigan C, Fischela JE, DeBaryshea BD, et al (1988) Accelerating language development through picture book reading. Developmental Psychology 24: 552–559.

[10] Peterson C, Jesso B, McAbe A (1999) Encouraging narratives in preschoolers: An intervention study. Journal of Child Language 26: 49–67. 10217889 10.1017/s0305000998003651

[11] Payne AC, Whitehurst GJ, Angell AL (1994) The role of home literacy environment in the development of language ability in preschool children from low-income families. Early Childhood Research Quarterly 9: 427–440.

[12] Snow CE, Ninio A (1986) The contracts of literacy: What children learn from learning to read books. In Teale WH, Sulzby E, editors. Emergent literacy: Writing and reading. Norwood, NJ: Ablex. pp. 116–138.

[13] Scarborough HS, Dobrich W (1994) On the efficacy of reading to preschoolers. Developmental Review 14: 245–302.

[14] Whitehurst GJ, Epstein JN, Angell AL, Paynea AC, Cronea DA, et al (1994) Outcomes of an emergent literacy intervention in Head Start. Journal of Educational Psychology 86: 542–555.

[15] Kuby P, Aldridge J, Snyder S (1994) Developmental progression of environmental print recognition in kindergarten children. Reading Psychology 15: 1–9.

[16] Masonheimer PE, Drum PA, Ehri LC (1984) Does environmental print identification lead children into word reading. Journal of Reading behavior 16: 257–271.

[17] McGee LM, Lomax RG, Head MH (1988) Young children's written language knowledge: What environmental and functional print reading reveals. Journal of Reading behavior 20: 99–118.

[18] Cronin V, Farrell D, Delaney M (1999) Environmental print and word reading. Journal of Research in Reading 22: 271–282.

[19] Dehaene S, Cohen L, Sigman M, Vinckier F (2005) The neural code for written words: a proposal. Trends in Cognitive Sciences 9: 335–341. 15951224 10.1016/j.tics.2005.05.004

[20] Perfetti C A, Liu Y, Tan LH (2005) The lexical constituency model: Some implications of research on Chinese for general theories of reading. Psychological Review 112: 43–59. 15631587 10.1037/0033-295X.112.1.43

[21] Yeh SL, Li JL (2002) Role of structure and component in judgments of visual similarity of Chinese characters. Journal of Experimental Psychology Human Perception and Performance 28: 933–947. 12190259

[22] Evans MA, Saint-Aubin J, Landry N (2009) Letter names and alphabet book reading by senior kindergarteners: An eye movement study. Child development 80: 1824–1841. 19930354 10.1111/j.1467-8624.2009.01370.x

[23] DeLoache JS, Chiong C, Sherman K, Islam N, Vanderborght M, et al (2010) Do Babies Learn From Baby Media? Psychological Science 21: 1570–1574. 20855901 10.1177/0956797610384145

[24] Yin L, Treiman R (2013) Naming writing in Mandarin-speaking children. Journal of Experimental Child Psychology 116: 199–215. 23831903 10.1016/j.jecp.2013.05.010

[25] Marulis LM, Neuman SB (2010) The Effects of Vocabulary Intervention on Young Children's Word Learning: A Meta-Analysis. Review of Educational Research 80: 300–335.

[26] Ho CS, Chan DW, Chung KK, Lee SH, Tsang SM (2007) In search of subtypes of Chinese developmental dyslexia. Journal of Experimental Child Psychology 97: 61–83. 17320097 10.1016/j.jecp.2007.01.002

[27] McBride-Chang C, Chow BW-Y, Zhong Y-P, Burgess S, Hayward W (2005) Chinese character acquisition and visual skills in two Chinese scripts. Reading and Writing 18: 99–128.

[28] Tan LH, Spinks JA, Eden GF, Perfetti CA, Siok WT (2005) Reading depends on writing, in Chinese. Proceedings of the National Academy of Sciences of the United States of America 102: 8781–8785. 15939871 10.1073/pnas.0503523102 PMC1150863

