# Supplementary material for: Do Preschool Children Learn to Read Words from Environmental Prints?
Source: PLoS One. 2014 Jan 22;9(1):e85745. doi: 10.1371/journal.pone.0085745 (PMC3899066; doi:10.1371/journal.pone.0085745)
Supplement: Table S1 — Results of ANOVAs for total scores of each item. (DOCX) [file pone.0085745.s001.docx]

# Supporting Information

**Table S1. Results of ANOVAs for total scores of each item.**

| Item | Version | Age | Version×Age |
| --- | --- | --- | --- |
| 肯德基 | *F*(1.94, 172.55)= 201.32^***^ | *F*(2, 89)= 12.40^***^ | *F*(3.88, 172.55)=3.15^*^ |
| 麦当劳 | *F*(1.86, 165.78)= 171.16^***^ | *F*(2, 89)= 11.60^***^ | *F*(3.73, 165.78)=3.40^*^ |
| 好多鱼 | *F*(2.14, 190.78)= 80.43^***^ | *F*(2, 89)= 12.33^***^ | *F*(4.29, 190.78)=2.15^*^ |
| 北京欢迎你 | *F*(1.97, 175.73)= 109.07^***^ | *F*(2, 89)= 24.08^***^ | *F*(3.95, 175.73)=2.77^*^ |

Note: 肯德基-KFC; 麦当劳-McDonalds; 好多鱼-Hao3 Duo1 Yu2; 北京欢迎你-Bei3 Jing1 Huan1 Ying2 Ni3; ^*^-*p*<.05, ^**^-*p*<.01, ^***^-*p*<.001
